# ATHLETIQUE: interest of an adapted physical activity program in patients with juvenile idiopathic arthritis: a feasibility and preliminary effectiveness study

**DOI:** 10.3389/fimmu.2023.1213799

**Published:** 2023-06-27

**Authors:** Stéphanie Py, Florine Maylié, Anne-Laure Parmentier, Chrystelle Vidal, Benjamin Cuinet, Fréderic Mauny, Anne Lohse, Eric Toussirot, Sagawa Yoshimasa, Nicolas Tordi, Delphine Binda, Claire Ballot-Schmit

**Affiliations:** ^1^ Inserm CIC 1431, CHU Besançon, Besançon, France; ^2^ Rheumatology Department, Nord Franche-Comté Hospital, Trevenans, France; ^3^ Rhumatologie, Pôle PACTE (Pathologies Aiguës Chroniques Transplantation Éducation), CHU Besançon, Besançon, France; ^4^ Département Universitaire de Thérapeutique, Université de Franche-Comté, Besançon, France; ^5^ UMR 1098 RIGHT, Inserm, Établissement Français du Sang, Université Franche-Comté, Besançon, France; ^6^ Laboratoire d’Exploration Fonctionnelle Clinique du Mouvement, CHU Besançon, Besançon, France; ^7^ UR 481 LINC Neurosciences and Cognition, Université de Franche-Comté, Besançon, France; ^8^ PEPITE, Platform Exercise Performance Health Innovation (EPHI), Université de Franche-Comté, Besançon, France; ^9^ Department of Paediatrics, CHU Besançon, Besançon, France

**Keywords:** juvenile idiopathic arthritis (JIA), adapted physical activity (APA) program, disease activity, juvenile arthritis disease activity score-27 (JADAS-27), pedometer watch

## Abstract

**Background:**

Juvenile Idiopathic Arthritis (JIA) is associated with joint inflammation, pain and limited joint mobility, impacting the practice of physical activities. Adapted Physical Activities (APA) are an increasingly used method of rehabilitation, but additional studies are needed to define the nature of the most appropriate physical activity for patients with JIA. The “ATHLETIQUE” project aims to evaluate the impact of a program integrating APA sessions with use of a pedometer watch, on disease activity in patients with JIA.

**Methods:**

This study will be a randomized, multicenter, open-label, controlled clinical trial with 2 parallel arms. The patients included in this study will be children and adolescents with JIA, aged 6 to 17 years. The experimental group (30 patients) will participate in an APA program for 3 months and will use a pedometer watch for one year. We will evaluate and compare the change in disease activity measurements (primary objective), fatigue, pain, quality of life, level of physical activity, functional capacities, and muscle strength (secondary objectives) after 14, 26 and 50 weeks. The control group (10 patients) will undergo the same evaluations as the experimental group but will not participate in the APA program and will not wear the pedometer watch.

**Expected results:**

The APA program may help to promote an active lifestyle with regular physical activity, preventing comorbidities and motor disability. Promising results on disease activity, functional capacities and quality of life would enable us to envisage a larger research program with a view to optimizing and assessing APA for children with JIA.

**Discussion:**

This study will be conducted in the short and medium-term, with one-year follow-up, including 3 months of APA sessions for the experimental group. The sessions proposed during the APA program will mainly be aerobic and bodyweight exercises. Furthermore, in contrast to previous studies on this topic, our study will integrate a novel element, namely the use of a pedometer watch. This watch will help to implement strategies to address motivation. This study aims to improve physical and mental well-being, provide a basis for the design of a larger study, and propose recommendations adapted to children with JIA.

**Trial registration:**

Registered with ClinicalTrials.gov under the number NCT05572424

## 1 Introduction

Juvenile idiopathic arthritis (JIA) is the most common cause of rheumatism in children in developed countries. Its prevalence ranges from 3.8 to 400/100,000 in Europe, and JIA affects about 4,000 patients in France (1). JIA encompasses a group of conditions that share common characteristics of arthritis, without a recognized etiology, of a least six weeks duration, and with onset of disease before the age of 16 years. Seven subtypes of JIA have been identified according to the International League for Rheumatology classification (2), of which two in particular are the most frequent, namely the oligoarticular and polyarticular forms, without rheumatoid factor. Together, these two forms account for two thirds of all JIA cases.

The symptoms of JIA are mainly swelling, stiffness and pain in the affected joints, as well as fatigue. The subsequent weakness and muscle atrophy result in reduced joint mobility, preventing some activities of daily life (3, 4). Some forms of JIA can have extra-articular symptoms such as ocular, cardiac, pulmonary, hematopoietic, or skin involvement (5). Symptoms and their severity are variable over time and among individuals. JIA persists in adulthood for half of the patients, and may result in motor disability, including difficulties with walking, prolonged standing or grasping. JIA has a negative impact on children’s quality of life, resulting in decreased physical well-being, depression, anxiety, and social isolation (6, 7). Quality of life is further impaired in severe forms, with increased pain, high disability, and a high number of actively inflamed joints.

To anticipate the possible complications related to disease progression, it is widely recommended to practice physical activity during childhood (6-12 years) and adolescence (13-17 years), preferably non-traumatic activity (e.g. swimming, walking, cycling …). Engaging in physical activity during these periods also helps to prevent and limit direct symptoms. However, there are no specific recommendations for physical activity in children with JIA. The following recommendations on physical activities issued by the World Health Organization (WHO) in 2010 are frequently used: “Children and youth aged 5–17 should accumulate at least 60 min of moderate- to vigorous-intensity physical activity daily. Amounts of physical activity greater than 60 min provide additional health benefits. Most of the daily physical activity should be aerobic. Vigorous-intensity activities should be incorporated, including those that strengthen muscle and bone, at least three times per week” (8).

Children with JIA present reduced functional capacities, practice less physical activity and sleep more than average children, regardless of disease level (9–12). In France, a study conducted from 2014 to 2016 among 1182 children showed that 41.8% of the children aged 6 to 17 years without diseases reached the WHO recommended level of 60 mins physical activity per day (13). Another study, conducted in the Netherlands in 30 patients with JIA and 106 controls showed that only 23% of children with JIA met the public health guidelines for physical activity (14). A follow-up study of physical activity by actimetry among 61 children with JIA versus 2055 healthy children confirmed that young patients with JIA were less active, and had lower physical capacity and fitness than healthy children (15).

The progressive decrease in the level of physical activity observed over time in patients with JIA could be associated with disease activity of JIA (in an inverse correlation), and with higher psychosocial stress related to the disease (16). The main barriers to the practice of physical activity are the severity of disease symptoms, the side effects of treatments, or the fear of worsening the disease (17). A vicious circle in inactivity and disease is initiated, leaving children with JIA more at risk of a sedentary lifestyle and cardiovascular complications in adulthood.

Many physical exercise programs have been evaluated in children with JIA, including mainly aquatic exercises, muscle strengthening, proprioception, endurance, and Pilates (18, 19). Studies of these programs have shown that the disease does not worsen with the practice of physical activity. On the contrary, beneficial effects of exercise seem to be observed in children with JIA, notably in terms of pain, physical and functional capacities (aerobic capacity, muscle strength, joint mobility, etc.) or on quality of life (12, 20). In addition, active children have a lower risk of obesity, which can increase joint loading.

Recently, a study among 23 children with JIA showed a direct correlation between hand grip strength and both quality of life (with an inverse correlation with disease activity), and functional capacity, with upper limb muscle strength measured by dynamometry (21). Significant strength gains were observed with balance training, skipping, core strengthening, and aquatic exercises. Pain is significantly reduced by programs combining aquatic exercises, muscle strengthening and Pilates (18).

To optimize prescriptions and recommendations for physical exercise for patients with JIA, additional studies are needed to define the nature of the physical activity that confers health benefits without exacerbating underlying inflammatory stress associated with the disease. Therefore, we propose to conduct the pilot “ATHLETIQUE” study, which aims to evaluate the impact of a program combining adapted physical activity (APA) sessions with the use of a pedometer watch, on disease activity in children with JIA, and to assess the effectiveness and safety of this intervention.

The study is expected to yield improvements in disease activity, physical fitness and weight control, an overall improvement in quality of life, and increased muscle strength, decreased pain, and increased functional capacities.

## 2 Methods and analysis

**Figure 1:**
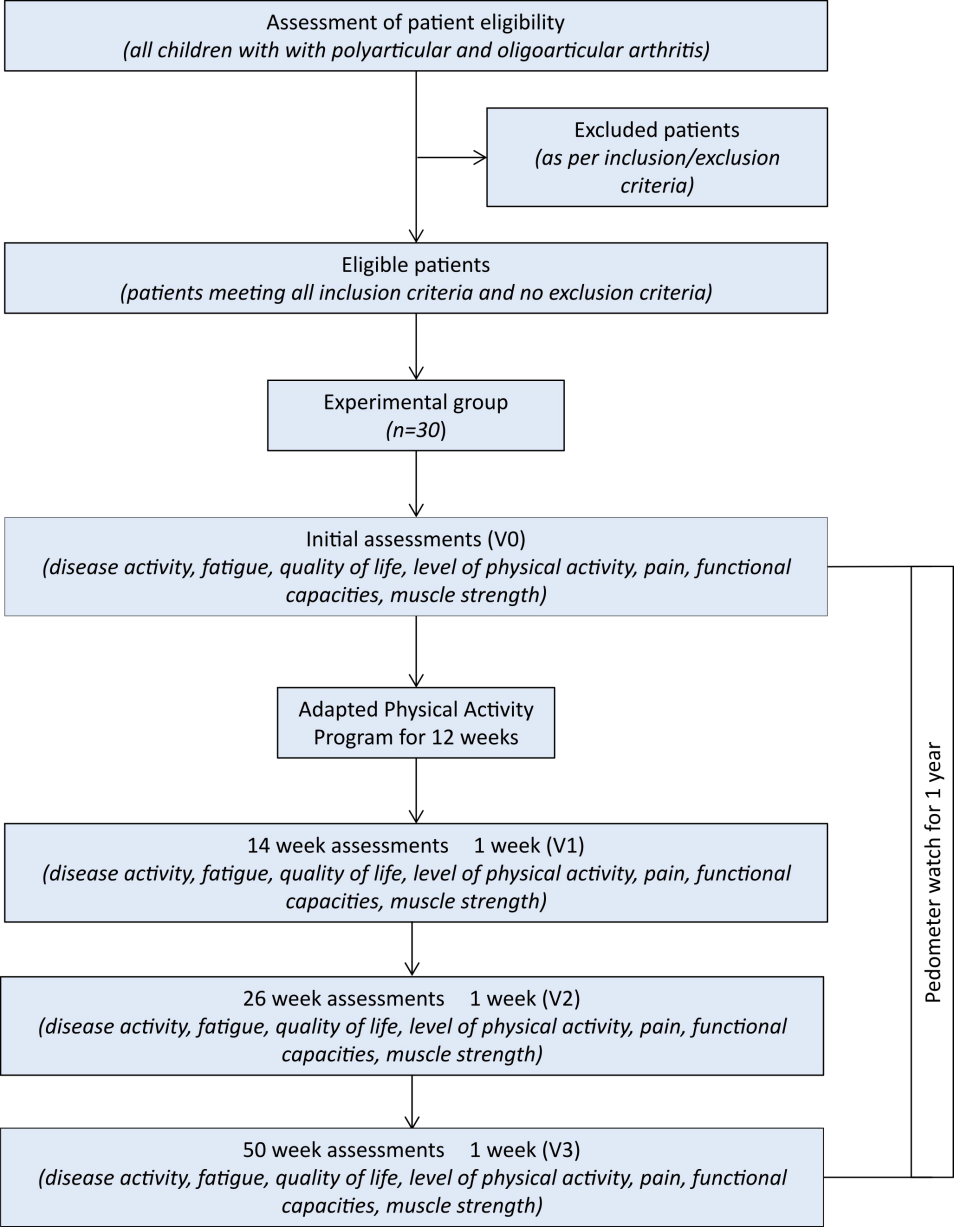
Flowchart of the study design.

The trial is a randomized controlled non-comparative interventional study including a group of patients undergoing an APA program for 12 weeks with the use of a pedometer watch (described in **Figure 1**) and a control group without the APA program. The study was registered with ClinicalTrials.gov (NCT05572424).

### 2.1 Patients

All patients aged between 6 and 17 years old, followed at the University Hospital of Besançon and the North Franche-Comté Hospital, for juvenile arthritis of the oligoarticular, polyarticular, psoriatic or enthesitis-related type, ongoing for more than one year, will be screened for potential eligibility. Patients with steady conventional or biologic treatment and who do not have any contraindication to physical activity (i.e. cardio-vascular disease, intra-articular injection in the previous month, peripheral joint with significant or advanced structural damage) will be eligible for recruitment. Potential candidates and their parents will be clearly informed about the study, and the children or adolescents will participate only after at least one of the parents provides consent. During the 3 months of APA sessions, included patients requiring an intra-articular injection or presenting physical inability to participate in the physical activity (e.g. due to injury), will be excluded. Patient inclusions will begin in the second half of 2023 and a total of 40 children and adolescents will be recruited during the study, 30 patients in the experimental group and 10 patients in the control group,.

### 2.2 Randomization

The randomization will be performed in a 3:1 (experimental:control) ratio into two groups, namely (1) the experimental group, who will participate in an APA program; and (2) a control group, who will not participate in the APA program. This study will be performed into two centers (University Hospital of Besançon and the North Franche-Comté Hospital, France) and recruitment will be competitive.

Randomization and data management will be performed using the CleanWeb business application (version 175.3.0), an electronic clinical trial management solution from Telemedicine Technologies (Boulogne-Billancourt, France).

### 2.3 Adapted physical activity program (only for experimental group)

The Adapted Physical Activity (APA) program will take place over twelve weeks, and will consist of two 40-minute sessions per week, for a total of 24 sessions. It will be designed by a professional in APA and a research engineer in technical sciences of physical activities and sports, based on the child’s profile, namely physical activity level, disease grade, pain intensity, joint mobility, fatigue, disease duration and personal preferences. The child will do the two weekly sessions in their own home: one of the two sessions will be broadcast live *via* online video conferencing, hosted by the APA professional. The second session will be done by the child in autonomy, using a sequence of different personalized exercises defined by the APA coach. Groups of up to five children will be formed based on age, for a maximum total of six groups. Each session will be broken down into three parts: warm-up (10 mins), the main core of the session, alternating between five to eight minutes of muscle strengthening, proprioception, and endurance exercises (total 20 mins) and a cool-down with stretching (10 mins). The proposed sessions alternate between continuous exercise sessions, with WODs (Workout Of the Day) such as AMRAP (As Many Repetitions As Possible) or For Time (number of rounds to perform in the shortest time possible), and intermittent exercise sessions with HIIT (High Intensity Interval Training). The proposed exercises are performed with bodyweight, and alternate between upper and lower limbs, i.e. full body sessions. The physical activity performed during the sessions will be quantified with a heart rate monitor (Kalenji HR300 model), provided to children at the inclusion visit and returned at the end of the 12week APA program. The target range of 60-70% of the maximum theoretical heart rate for each child will be respected (11, 12). The content of the sessions will be adapted over the course of the program according to each child’s progress.

### 2.4 Outcome measures

**Figure 2:**
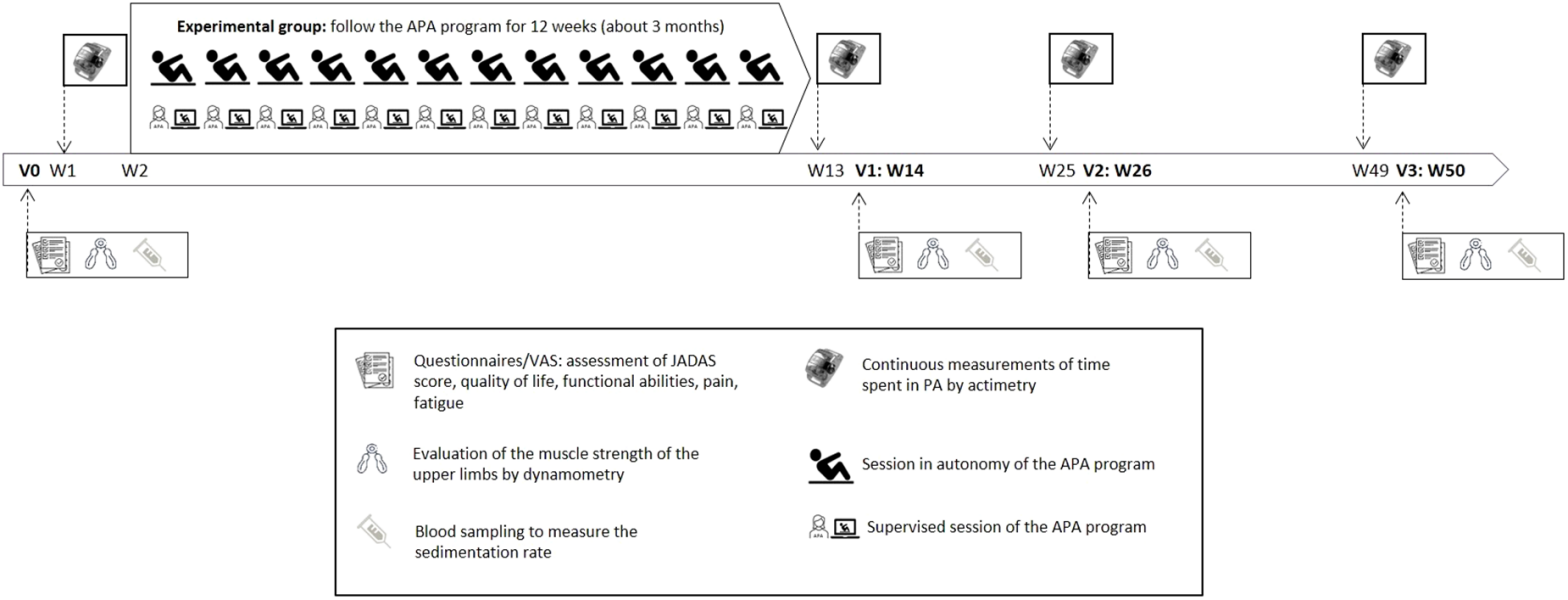
Conduct of the study.

Assessments performed at the inclusion visit (V0), at 14 ± 1 weeks (V1), 26 ± 1 weeks (V2) and 50 ± 1 weeks (V3), will focus on disease activity, fatigue, quality of life, functional capacity, pain, and muscle strength (**Figure 2**). In addition, all patients included will be asked to wear an actimeter (Actigraph model wGT3X-BT) for one week after the initial assessment and the week before the other assessments, to measure their level of physical activity. Patients in the experimental group will also be given a non-connected pedometer watch and activity tracker (Willful, Shenzhen, China), which they will wear for one year, starting from the initial assessment (V0), through to the final assessment (V3). This watch will provide the number of steps taken during the entire study period. Children or their parents, depending on their age, will collect this data in a follow-up booklet, every day of one week per month for twelve months.

#### 2.4.1 Primary outcome: disease activity

Disease activity will be assessed by the change in Juvenile Arthritis Disease Activity Score (JADAS-27) between the initial assessment (V0) and V1 (week 14).

The JADAS-27 score consists of four parts, three of which are directly related to the disease, and the fourth to patient well-being, as follows:

1. The number of inflammatory joints among 27 identified,
2. A physician assessment of clinical disease activity on a 10-point visual analogue scale (VAS),
3. An assessment of patient’s well-being by a 10-points VAS, scored by the patient or a parent,
4. The normalized erythrocyte sedimentation rate (0 to 10).

JADAS-27 scores range from 0 to 57. The higher the score, the more active the disease. It is expected that the JADAS-27 score will be reduced by 2.7 points between the initial assessment (V0) and 14 weeks (V1 - post APA program). This minimal clinically important difference (MCID) corresponds to the decrease needed to go from low clinical impairment (22) before the APA sessions (score=2.7) to no disease activity (score=0).

#### 2.4.2 Secondary outcomes:

Evaluation of the effect of the program on disease activity assessed by the change of JADAS-27 score compare to baseline, at 26 and 50 weeks ± 1 week.

Evaluation of the physical activity level: The difference in the average level of physical activity will be assessed by continuous measures (over 7 days) of the time spent in MVPA (Moderate to Vigorous Physical Activity) *via* actimetry (count/min) (for both groups) and the number of steps estimated by the pedometer watch (only in the experimental group).

Actimetry measurements will be performed before the start of the intervention to determine a value for V0, at V1-1 week, V2-1 week and V3-1 week, and pedometric measurements will be performed throughout the study but collected one week per month to estimate a monthly value.

Quantification of the difference in muscle strength (expressed in Newtons) by evaluation of the grip strength, using a dynamometer at weeks 14, 26 and 50 ± 1 week.

Quality of life, functional capacities, pain and fatigue at weeks 14, 26 and 50 ± 1 week: a) Quality of life will be assessed by the difference over baseline on the score obtained on the PedsQL (Pediatric Quality of Life Inventory) tool (questionnaire and VAS). The PedsQL MCID is 4.4 points (23, 24), b) Functional capacity and pain will be assessed by the CHAQ-38 (Childhood Health Assessment Questionnaire-38) tool (questionnaire and VAS). The CHAQ MCID for improvement is -0.188 at most, while the MCID for worsening is at most +0.125 (25), c) Fatigue among those participating in the APA program will be assessed by the difference over baseline in the score obtained in the PedsQL-Multidimensional Fatigue Scale (MFS) questionnaire.

Assessment of the feasibility of the program: The feasibility endpoint will focus on participation, completion, adherence, compliance, and watch use time. Participation is defined as the proportion of subjects who agree to participate in the study among eligible patients. Completion will be assessed by the proportion of subjects who complete the full program, and by the proportion of subjects who complete all the exercise sessions. Adherence is defined as the proportion of sessions attended by each subject. Compliance is the proportion of prescribed exercise actually performed during the session. These four criteria will be assessed during the period of the APA sessions, from week 2 to week 13. Finally, watch use is defined as the amount of time the pedometer watch was used over the duration of the study.

### 2.5 Statistical justification of the number of patients to be included

The calculation of the number of participants required is based on a preliminary estimate of the effectiveness of the program, based on the following assumptions: a mean decrease in the JADAS-27 score of 2.7 points, a standard deviation 4.6 points [based on a previous descriptive study conducted on 24 patients at the University Hospital of Besançon (26)], alpha risk of 0.05; 80% power, bilateral testing The estimated number of participants in the experimental group is 25. Allowing an additional 5 patients to cater for missing or lost data, the number of subjects to be included is set at 30. The calculation is based on a “medium to moderate” effect size of 0.6 points. In the absence of previous studies, and taking into account the clinicians’ experience, these assumptions appear to be plausible

Ten additional patients will be included in a control group (without APA). This group will allow to estimate the “natural” progression of the primary outcome without any APA program (control); Follow-up data (excluding activity assessment) will also be collected on this 10-patient group.

Randomization will be performed in a 3:1 (experimental:control) ratio according to the design of the study.

The study methodology and statistical analyses are managed by the research methods unit (uMETh) of the clinical investigation center, University Hospital of Besancon.

### 2.6 Data analysis

Quantitative data will be described as means and standard deviation, or median and range. Qualitative variables will be described as number and percentage, for each response category. No statistical test will be performed to compare the parameters between the two groups (no comparative study).

The mean difference in the primary endpoint between V0 and V1 (with 95% confidence interval) will be calculated in the two groups. Paired t-tests or Wilcoxon signed ranks tests will be used to test the hypothesis of an effect of the APA program in the intervention group.

The secondary endpoint analysis:

The mean difference (and the 95% confidence interval) in the level of physical activity, expressed in Counts/min, between V0 and V1 will be calculated. The physical activity level will be presented graphically for each measurement time and for each group.

The number of meters walked on average per month will be presented graphically for each measurement time, in the experimental arm only.

Average scores on the JADAS-27, CHAQ-38 (9 activity items evaluated and score of functional incapacity, pain, and the parent or patient global assessment of arthritis), Peds-QL (sub-score physical and psychological health) and PedsQL-MFS (sub-score sleep quality, general and cognitive fatigue) with standard deviations will be presented graphically for each measurement time (V0-V1-V2-V3) for each group, and also by age group (6-7 years, 8-12 years, 13-17 years) within each group.

As an exploratory basis, if the data are amenable, univariable linear regression model will be used to test the variables potentially associated with progression of disease activity, physical activity, and quality of life (age, body mass index, number of swollen joints).

The percentage of patients accepting to participate to the study among the eligible patients, the percentage of patients completing the program, the percentage of patients completing exercises sessions will be calculated.

The mean percentage of sessions in which patients participate and the mean percentage of prescribed exercises performed during the session will be also calculated.

## 3 Discussion

Particular attention will be paid to the period chosen for the beginning of this study in order to avoid bias. To this end, the program will be implemented after the summer vacation, at a time when children are available, and to be representative of usual activity. The study will take place over a period of 50 weeks ± 1 week, including 12 weeks with APA sessions. The few studies performed on this subject to date conducted the APA program over 10 to 48 weeks (27), with a median length of 12 weeks. A 12-week period seems to be sufficient to offer an APA program, at a rate of two sessions per week, and thus obtain significant initial results. However, previous studies on physical activity in JIA have been performed in the short term (28). It would be interesting to observe the beneficial effects of physical activity over the medium or long-term. For this reason, we decided to conduct this study over 1 year, with 4 time assessments using actimetry. Actimetry is an objective method of measure compared with self-report questionnaires, and is validated for monitoring physical activity in healthy children as well as in children with chronic diseases. However, it is not widely used in children with JIA (29, 30).

A recent review of literature on this subject (28) reported a limited interest of strategies to address motivation and develop healthy behaviors at an early stage. Therefore, in addition to the APA sessions, it seems helpful to include a novel aspect in the care of these children, namely the use of a pedometer watch throughout the study. The participants in the experimental group will wear the pedometer watch for 1 year, not only to quantify the time spent in activity, but also to increase the motivation of these children to engage in physical activity. The pedometer watch will make it possible to identify when motivation is highest and lowest in relation to the APA sessions, and will also enable the children to become aware of their daily activity, in terms of the number of steps and the time spent being active, notably whether it is sufficient or not. They will then have the possibility to perform self-evaluation and self-adaptation of their lifestyle to correspond with the WHO recommendations.

Concerning the APA sessions, the exercises proposed will be aerobic exercises, for physical endurance, and bodyweight training, for physical strength, as we expect greater improvement in these two skills. The progress in terms of physical strength (evaluated by dynamometry) should be of lesser magnitude than the progress in physical endurance (qualitative criterion evaluated over APA sessions), due to the choice to propose aerobic and bodyweight exercises. Aerobic exercises develop physical endurance whereas body weight exercises focus on physical strength (31). However, body weight exercises do not lead to rapid muscle mass gain and correlated physical strength, thus a lower speed of progression of the physical strength than the endurance should be observed. Nevertheless, we expect the most marked results in terms of physical and mental well-being (assessed by PedsQL, PedsQL-MFS and CHAQ-38) and disease activity (assessed by JADAS-27).

**Figure 3:**
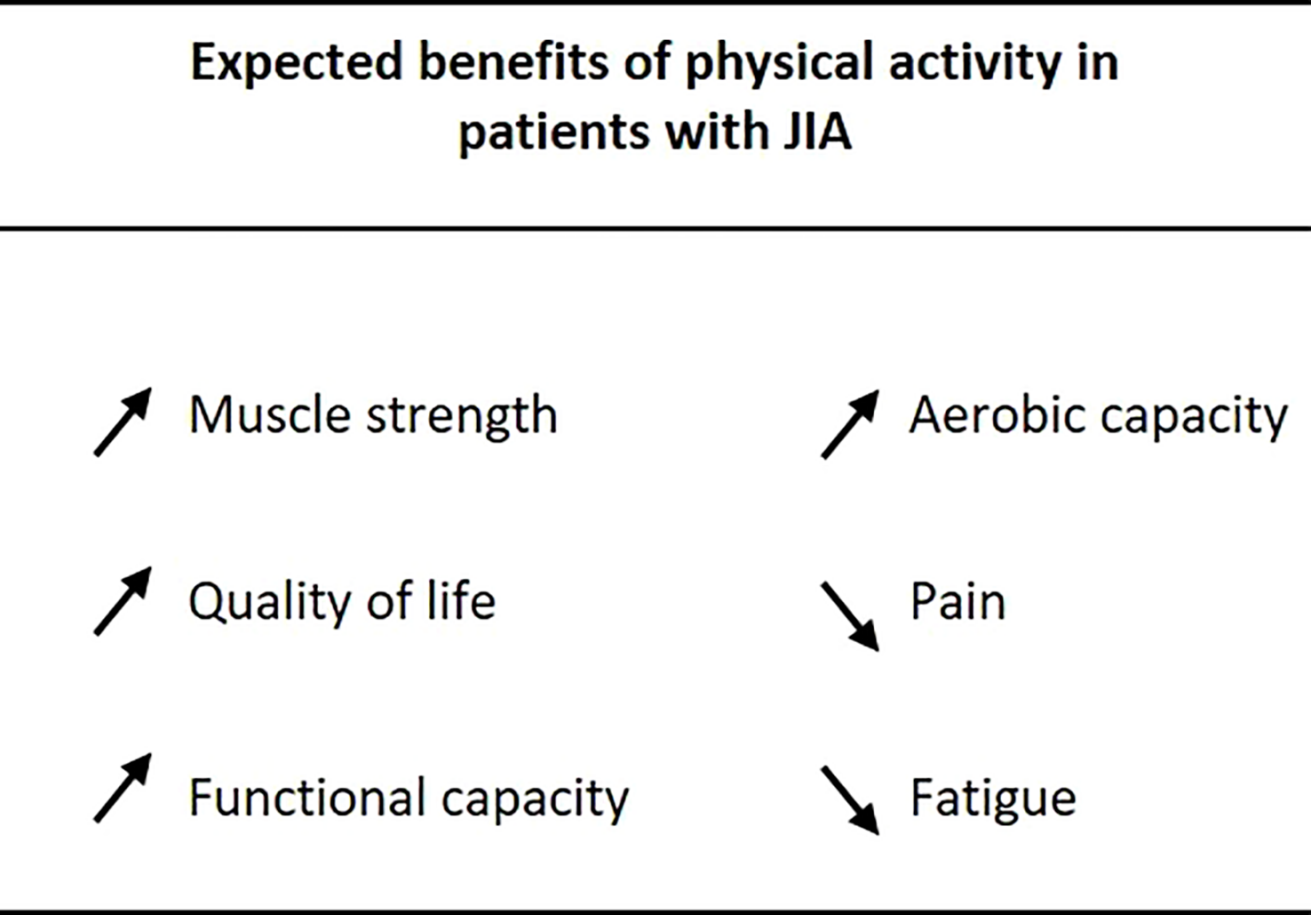
Expected benefits of physical activity in patients with JIA.

In addition to the variation in clinical parameters (quality of life, muscle strength, pain, functional capacity) as shown in **Figure 3**, an enhanced understanding of APA by children and their parents is also expected. Children will engage in sports sessions at home, on their own, following personalized advice. They will also participate in group APA sessions, supervised by a professional APA *via* an online video conference, thus limiting the social isolation that is often inherent to the disease. This program could constitute a trigger for a lasting commitment to the practice of physical activity in autonomy. In the long term, promoting physical activity in young patients could help to prevent or delay the onset of motor disability, and of comorbidities related to a sedentary lifestyle in adulthood (diabetes, obesity, cardio-vascular diseases, bone damage, etc.). We hope to be able to apply and extend the results obtained in this study to perform a larger clinical trial in patients with JIA, with a view to issuing proposals for personalized care through APA sessions together with the use of a pedometer watch. This study will provide baseline data to be used as a foundation for a larger study for better care of patients with JIA, in terms of physical activity, and to propose recommendations adapted to children with JIA, since the recommendations of the WHO are for the general population of children (60 mins of moderate to intense physical activity per day).

## Data availability statement

The original contributions presented in the study are included in the article/supplementary materials. Further inquiries can be directed to the corresponding author.

## Ethics statement

The study was approved by the Ethics Committee “Comité de Protection des Personnes Est-III” under the number 2022-A01118-35 on June 09, 2022. At least one parent of each patient will provide written informed consent before inclusion. The University Hospital of Besancon is the trial sponsor. Results of the study will be submitted for publication in a peer-reviewed international medical journal.

## Author contributions

SP and FlM contributed equally to the writing of the entire protocol. A-LP, CV and FrM brought a global expertise in methodology and particularly contributed to the drafting of the parts entitled Randmisation, Statistical justification of the number of patients to be included and Data analysis. BC, FlM, NT and YS Junior brought their expertise in the field of adapted physical activity and particularly contributed to the writing of the sections Adapted Physical Activity Program and Outcomes. AL, ET and CB-S brought their medical expertise and particularly contributed to the Introduction, Discussion and Outcomes sections. SP, DB and ET brought their expertise in clinical research and contributed to the drafting of the protocol in its entirety. All authors contributed to the article and approved the submitted version.
